# Dynamic Population Distribution and Perceived Impact Area of the Tibet Dingri *M*_S_6.8 Earthquake Based on Mobile Phone Location Data

**DOI:** 10.3390/s26020457

**Published:** 2026-01-09

**Authors:** Huayue Li, Chaoxu Xia, Yunzhi Zhang, Yahui Chen, Wenhua Qi, Fan Yang, Xiaoshan Wang

**Affiliations:** 1 Institute of Geology, China Earthquake Administration, Beijing 100029, China; lihy@seis.ac.cn (H.L.); qiwenhua@ies.ac.cn (W.Q.); 2 China Earthquake Networks Center, Beijing 100045, China; zhangyunzhi@lreis.ac.cn (Y.Z.); chenyahui@seis.ac.cn (Y.C.); 3 Hebei Earthquake Agency, Shijiazhuang 050021, China

**Keywords:** mobile phone location data, earthquake emergency response, dynamic population distribution, perceived impact area

## Abstract

Based on the collected mobile phone location data, this paper analyzes changes in four mobile location-based indicators and their spatiotemporal distribution characteristics before and after the earthquake, summarizing crowd movement patterns and communication behaviors after the *M*_S_6.8 Dingri earthquake. By comparing natural neighbor interpolation and Thiessen polygon interpolation methods, we explore novel rapid assessment approaches for earthquake perception ranges, combined with actual seismic intensity maps. The results indicate an uneven distribution of population and differing dynamics in mobile phone signal activity. This reflects different behavioral patterns and the potential perceived extent of the earthquake. Within 50 km of the epicenter, all four indicators showed varying degrees of decline post-earthquake, while areas beyond 100 km exhibited short-term surges, reflecting differentiated behavioral responses based on seismic impact severity. In areas experiencing strong shaking, risk avoidance behavior predominated, while in areas where shaking was noticeable but less severe, communication behavior was more prominent. Mobile data decline zones showed high spatial correlation with intensity VIII+ regions, proving their effectiveness as rapid indicators for identifying strongly affected areas. Notably, mobile location data enabled accurate identification of strongly affected zones within 30 min post-earthquake.

## 1. Introduction

Earthquake emergency rescue, as a critical component of the earthquake prevention and disaster mitigation system, directly affects the reduction in casualties and economic losses through its operational efficiency [1]. As the most immediately impacted population, survivors’ mobility and distribution characteristics are closely linked to earthquake emergency response. Accurate population distribution and mobility data are vital foundational resources for post-disaster decision-making [2]. Traditional population distribution datasets, which rely on census and sampling surveys, are typically aggregated by administrative divisions and updated every five years. These datasets suffer from long update cycles, low spatial resolution, and an inability to reflect small-scale population mobility, making them inadequate for rapidly capturing real-time dynamic population distribution during the post-disaster “black box period” [3,4]. If precise population data are unavailable during the initial earthquake emergency phase, remote areas often plagued by communication failures and road damage may experience delayed disaster reporting. This can lead to rescue efforts being disproportionately allocated to urban zones with clearer situational information, resulting in a mismatch between resource deployment and actual needs. However, mobile phone location data, characterized by high spatiotemporal resolution, extensive coverage, and strong real-time capability, offer a novel approach for rapidly acquiring post-earthquake population distribution. This helps address the critical challenge of real-time disaster assessment during the “black box period” [5,6].

Previous studies highlight the potential of mobile phone data in disaster management cycles. Pre-disaster, historical trajectory data can predict high-risk population aggregation zones and optimize evacuation plans [7,8]. During disasters, multi-source data fusion enables real-time victim localization and dynamic monitoring of population movements [9]. Post-disaster, mobile data aids in assessing resettlement needs and tracking population return trends [10]. Scholars have conducted diverse studies leveraging mobile phone location data for disaster research. Following the 2010 Haiti earthquake, Lu pioneered the use of mobile operator data to reveal predictable patterns in post-earthquake population mobility. During the 2011 Great East Japan Earthquake, Kanno developed a high-risk evacuation route assessment model using geotagged Twitter data [11]. After the 2015 Nepal earthquake, Wilson’s team analyzed 12 million call records to estimate post-disaster population displacement. In the context of China’s 2017 Jiuzhaigou MS7.0 earthquake, Li [12] quantified spatiotemporal population distribution dynamics through 24 h continuous mobile positioning data, establishing a framework for rapid post-disaster population monitoring. Pang [13] further advanced the field by conducting granular analyses of mobile location data characteristics, identifying differential sensitivities of distinct data types to seismic events. After Hurricane Maria in 2017, Takahiro Yabe [14] examined inter-city dependencies across 78 Puerto Rican counties via mobile datasets, advocating for enhanced cross-regional collaboration in disaster preparedness alongside infrastructure hardening. Most recently, Yang [15] investigated crowd evacuation processes and emergency response capabilities during the 2021 Henan catastrophic floods by comparing 24 h pre- and post-disaster mobile trajectories, providing critical references for optimizing urban flood emergency protocols.

Existing research demonstrates the multi-phase applicability of mobile phone location data across the disaster management cycle. During the pre-disaster preparedness phase, historical trajectory data enables predictive identification of high-risk population aggregation zones. Population density analysis in these zones supports evacuation protocol development [7] and facilitates evacuation route optimization modeling [8]. In the disaster response phase, real-time victim localization is achieved through multi-source data fusion, enabling dynamic monitoring of population clustering, migration patterns, and evacuation routes to optimize rescue resource allocation [16,17]. For post-disaster recovery, population behavior analysis enables assessment of shelter demand capacity and spatiotemporal estimation of displaced populations [10]. Longitudinal tracking of population return trends further informs resettlement planning and reconstruction strategies [9]. The convergence of Geographic Information Systems (GIS) and artificial intelligence has catalyzed a paradigm shift in disaster management—from experience-based to data-driven decision-making [6]. Practical implementations are already emerging: Jiangxi Province’s Emergency Management Department has integrated real-time population monitoring through 858,000 telecommunication base stations across three major operators, enabling immediate post-disaster population distribution mapping [18].

This study investigates the Dingri earthquake through comparative analysis of mobile data variations and spatiotemporal distribution characteristics before and after the seismic event. By examining post-earthquake population movement and distribution patterns, we employ Natural Neighbor Interpolation and Thiessen Polygon methods to explore methodologies for assessing the perceived impact area. The research establishes a theoretical-technical framework supporting three critical post-disaster applications: (1) dynamic population distribution sensing, (2) behavioral pattern analysis of affected populations, and (3) rapid evaluation of seismic perception zones.

## 2. The Dingri Earthquake

**Figure 1:**
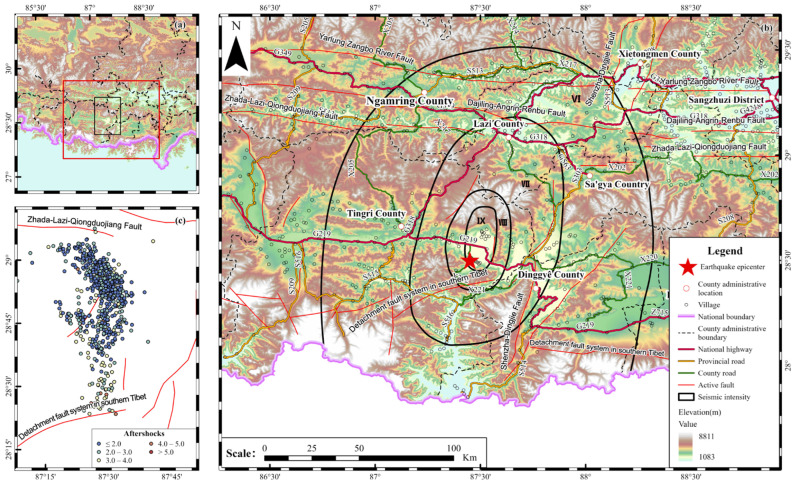
Tectonics and seismic intensity distribution map around the Dingri earthquake. (**a**) Topography around the study area. The red box indicates the coverage of (**b**), and the black box indicates the coverage of (**c**). (**b**) Active faults and seismic intensity distribution in the study area. The red pentagram marks the epicenter of the *M*_S_6.8 Dingri earthquake. (**c**) Aftershock distribution of the *M*_S_6.8 Dingri earthquake. Red lines represent active faults, and colored circles show the distribution of aftershocks within 48 h after the mainshock. Roman numerals denote macroseismic intensity from degree VI to degree IX [24].

At 9:05 (Beijing time) on 7 January 2025, a magnitude *M*_S_6.8 earthquake struck Dingri County, Shigatse City, Tibet Autonomous Region (28.50° N, 87.45° E), with a focal depth of 10 km. Located at the frontal collision zone of the Indian Plate and Eurasian Plate, Dingri lies within the western segment of the Himalayan seismic belt. The main shock’s epicenter occurred in the Dingmucuo graben, in the western segment of the southern Xainza Dinggye rift zone in the southern Tibetan Plateau. The affected region includes Quluo and Cuoguo town, characterized by east–west trending normal faults within Triassic-Jurassic strata, and Changsuo town, situated near east–west trending Jurassic thrust faults and the north–south trending Dengmucuo normal fault. The earthquake exhibited a typical normal-faulting mechanism [19,20,21], generating a maximum macroseismic intensity of IX (Figure 1) and a 26 km surface rupture zone. The disaster impacted 200,000 people across seven counties (Dingri, Lazi, Sajia, Dingjie, etc.), with 139,000 evacuated, 126 fatalities, 26,900 collapsed buildings, 215,000 damaged structures, and direct economic losses totaling 8.945 billion yuan [22,23]. The VI-degree intensity zone, characterized by an average elevation of 4923 m, posed significant challenges to emergency rescue operations due to high-altitude terrain and fragile infrastructure.

## 3. Materials and Methods

### 3.1. Mobile Phone Location Data

The widespread adoption of smart mobile devices in China has led to the installation of numerous third-party applications. These applications rely on push notification services enabled by Push Software Development Kits (SDKs) provided by mobile service providers. These SDKs, compliant with data security standards, collection scopes, and transmission protocols, utilize built-in functional modules to periodically collect user-authorized geolocation data. This data encompasses device identifiers, GPS coordinates, Wi-Fi signatures, cellular base station logs, and network connectivity metadata. After encryption, these multi-source data streams inputs are aggregated into structured mobile location datasets.

The datasets employ Geohash6 encoding with four signaling indicators whose technical specifications are summarized in Table 1. (1) Active Base Stations: base stations scanned and periodically reported by mobile devices. (2) Active Wi-Fi Hotspots: Wi-Fi hotspots scanned and periodically reported by mobile devices. (3) Mobile Devices: mobile devices that obtain services through multiple positioning methods. (4) Wi-Fi-Connected Devices: mobile devices connected to Wi-Fi hotspots. These signaling indicators provide high-resolution insights into population mobility patterns during disasters.

**Figure 2:**
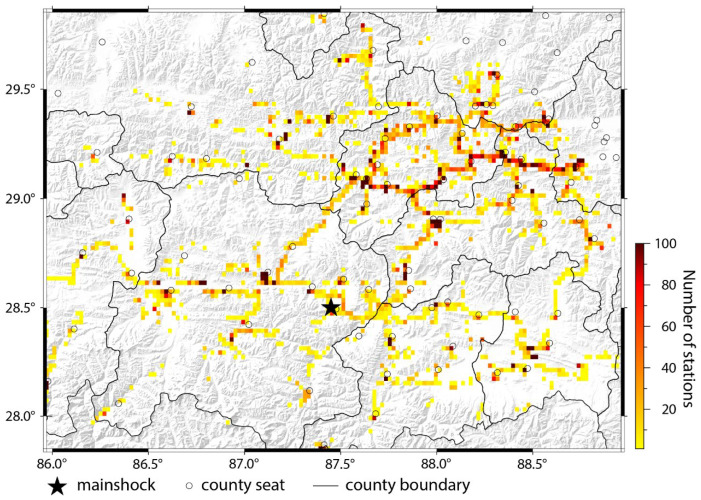
Distribution of mobile phone location data. Data partitioned into 0.02° × 0.02° grids via spatial scanning, with each grid cell containing the sum of mobile signaling indicators within its bounds. Grid coloration visualizes the numbers of mobile signaling indicators, where darker shades indicate higher data submission counts within individual grids.

The mobile location dataset covers a 150 km radius from the epicenter, spanning 38 h from 9:00 on 6 January to 1:00 on 8 January. It comprises 146,123 records. Each Geohash6 grid cell contains central coordinates (decoded latitude/longitude), signaling indicator counts (the numbers of mobile devices/active base stations/active Wi-Fi hotspots/Wi-Fi-connected devices per grid) and timestamp (minute-level precision; see Table 1). Data sample exemplified in Table 2. Due to the limited spatial resolution of Geohash6 grids (approximately 1.2 km × 0.6 km), which represent relatively small rectangular areas, we employed a spatial grid scanning approach with a step size of 0.02° longitude × 0.02° latitude to better illustrate the spatial distribution characteristics of the dataset cover the study area. For each grid cell, we aggregated the corresponding data values. This methodology enables effective macroscopic visualization of disaster-affected area data distribution patterns. The result is shown in Figure 2.

### 3.2. Methodology

Historical earthquake disaster investigations demonstrate that seismic events significantly impact communication systems within affected regions, causing distinct variations in mobile location indicators. By analyzing pre- and post-earthquake changes, this study identifies patterns in post-disaster human mobility and delineates the earthquake’s perceived impact area. To model population density in the affected area, we employ Gaussian kernel density estimation (KDE). Mobile signaling data in disaster zones is fragmented as the population is discretely distributed. This non-parametric method reconstructs continuous density surfaces from fragmented mobile signaling data. The technique centers a Gaussian kernel function at each data point and averaging the superposition of all kernel functions. The kernel function employed in this method is the standard Gaussian function:

(1) $$K(u)=\frac{1}{\sqrt{2\pi }}e^{-\frac{1}{2}u^{2}},$$

where $u=\frac{\left\|x-x_{i}\right\|}{h}$, with $\left\|x-x_{i}\right\|$ representing the distance between the target point *x* and the data point *x_i_*, and *h* denoting the bandwidth—a critical parameter controlling the width of the kernel function. A larger bandwidth yields smoother density estimates but may obscure fine-scale features, whereas a smaller bandwidth preserves finer details but risks amplifying noise.

The spatial distribution of earthquake-induced hazards demonstrates significant spatial dependence. To quantify spatiotemporal data variation rates, this study applies comparative interpolation analyses using Thiessen polygons and natural neighbor methods, establishing a dual-method framework for assessing the Dingri earthquake’s perceived impact range. Thiessen polygon interpolation, is a discrete method based on spatial proximity, partitions the study area into polygonal regions where each contains a single sample point, establishing explicit “proximal zones of influence” wherein all locations are closer to their associated sample point than to others. Its interpolated boundaries are uniquely determined by the spatial distribution of sample points, requiring no predefined data distribution patterns. This method generates discrete Voronoi tessellation through computationally efficient algorithms characterized by logical simplicity. However, it demonstrates significant spatial continuity limitations. Conversely, natural neighbor interpolation employs a continuous weighted approach based on Thiessen polygon geometry. It calculates spatial weighting factors determined by the contributing Voronoi cell area of neighboring samples to generate continuous gradient surfaces that represent signaling variation rates. The results manifest smooth transitional gradients across local neighborhoods, effectively addressing the continuity constraints inherent to Thiessen polygons. However, it requires dynamic computation of neighbor weights—a process incurring substantial computational overhead. To enhance the precision of post-disaster impact area, we conducted comparative interpolation analysis.

Using grids based on Geohash level 6 encoding as the basic spatial unit, this study analyzes post-earthquake human activity and the actual felt area by comparing data changes before and after the earthquake. In this paper, the average beacon value from the 2 h before the earthquake is used as the pre-event baseline. Signal variation rates are then calculated by comparing data from different post-earthquake time windows—such as 5 min, 10 min, and 30 min—against this baseline. Let *N_i_* denote the indicators value recorded in a single grid cell at *i*-minute intervals post-earthquake, and *K_j_* represent the pre-seismic baseline—the average indicators value within the same grid over *j* minutes prior to the event. The data variation rate (*R*) is calculated as

(2) $$R=\;\frac{N_{i\;}-K_{j}}{K_{j}}\times 100\%$$

Positive *R* indicates increased indicator values in the grid cell post-earthquake, typically reflecting population influx (e.g., evacuation clustering or rescue team deployment). Negative *R* signifies decreased indicators values, suggesting population outflow (e.g., rapid evacuation or infrastructure disruption).

## 4. Population Distribution Analysis

### 4.1. Pre-Earthquake Population Distribution

**Figure 3:**
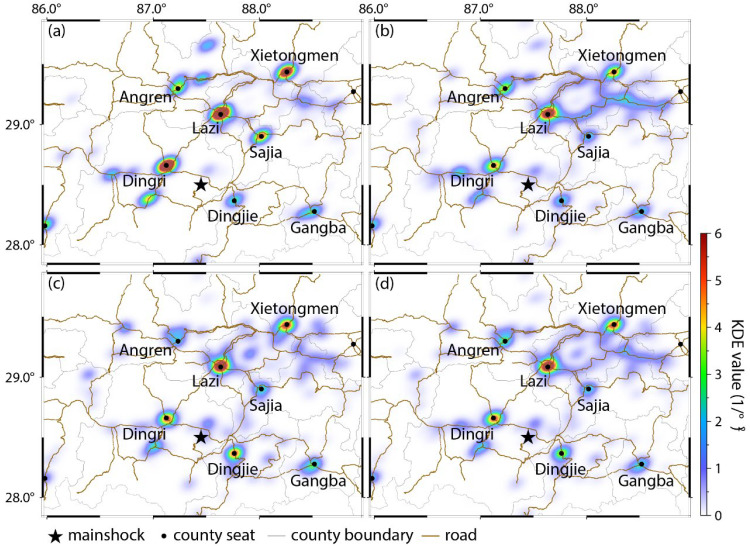
Distribution of signaling indicators. (**a**) Active Wi-Fi hotspots; (**b**) Active base stations; (**c**) Wi-Fi-connected devices; (**d**) Mobile devices. Grid coloring visualizes the reported value of the mobile signaling indicator, where the closer the color is to the warm color, the higher the indicator density.

To establish baseline population distribution patterns, mobile location data within the study area from 2 h pre-earthquake period were analyzed using Gaussian kernel density estimation. The closer the color on the color bar is to warm color, the higher the indicator density. As shown in Figure 3, all indicators consistently indicate that low signal activity near the epicenter, with minimal population distribution in northern Changsuo town. High-density clusters concentrated in Dingri, Lazi, Xietongmen, Dingjie, and Ngamring counties, exhibiting dense signals and substantial population aggregation. Linear alignment of signals locations along highways, reflecting elevated population density along major transportation corridors. Extremely sparse signals in the southeastern plateau region adjacent to the epicenter, suggesting minimal permanent settlement. Mobile data volume strongly correlates with regional development levels: (1) Economically advanced areas with robust road infrastructure exhibit higher population density and richer data coverage. (2) Peripheral mountainous zones exhibit data scarcity due to low habitation density and limited telecommunication coverage.

### 4.2. Post-Earthquake Population Distribution

**Figure 4:**
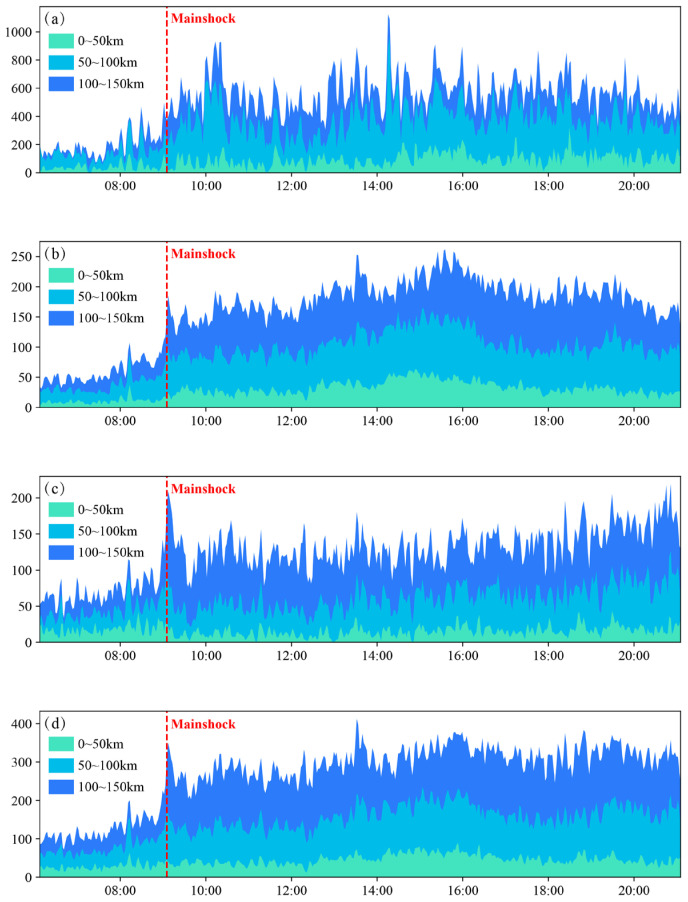
Temporal variation in signaling indicators. (**a**) Active Wi-Fi hotspots; (**b**) Active base stations; (**c**) Wi-Fi-connected devices; (**d**) Mobile devices.

We extracted and analyzed aggregate counts of four indicators (active Wi-Fi hotspots, active base stations, mobile devices, and Wi-Fi-connected devices) within three concentric zones (0–50 km, 50–100 km, 100–150 km from the epicenter) at 1 min temporal resolution (Figure 4). The data reveal significant pre-seismic spatial heterogeneity between 5:00 and 9:07. Within 50 km zone exhibited the lowest total signal counts (light green area), reflecting sparse population density near the epicenter. 100–150 km from the epicenter showed peak signal intensity (dark bule area), indicating major population centers 100–150 km from the epicenter. All zones demonstrated synchronized counts increases starting at 8:30—closely aligned with local dawn (twilight at 8:30; sunrise at 8:59). This pattern suggests intensified human activity correlated with post-dawn activity resumption.

Following the seismic event (9:07, red line), distinct zonal response characteristics emerged: within 50 km from the epicenter (light green area), the quantities of signaling indicators exhibited varying degrees of decline within 30 min after the mainshock, potentially related to direct seismic impacts such as power outages, base station failures, and population displacement. Within 50–100 km (blue area), indicators surged within 5 min, dominated by spikes in active Wi-Fi hotpots, active base stations, and mobile devices—indicative of emergency communication behaviors among residents. The region between 100 km and 150 km (dark bule area), encompassing densely populated urban areas, exhibited the most pronounced post-seismic response in mobile signaling activity.

Taking Wi-Fi-connected devices as an example (Figure 4c), the communication network experienced an immediate surge of signaling requests following the earthquake, forming a characteristic “signaling tsunami” phenomenon. This pattern indicates that despite experiencing perceptible ground motion, the region’s critical infrastructure remained largely functional, facilitating prompt information-seeking and social connectivity behaviors among residents. The mobile signaling volume began declining from its peak value within 10 min post-earthquake, likely attributable to residents completing critical safety verification (including seismic information confirmation and family status checks) before physically evacuating Wi-Fi coverage zones for protective actions. Thirty minutes later, all indicators maintained sustained high activity levels. This phenomenon is likely attributable to the progressive arrival of rescue teams and the initiation of rescue operations.

The observed spatial response gradient reveals a significant negative correlation between seismic impact intensity (proximity to epicenter) and population density distribution derived from mobile location data. Post-disaster communication intensity increased proportionally with greater epicentral distance. Exploratory analysis of these findings demonstrates that spatially distributed mobile data provides novel data-driven support for rapid seismic impact field assessment in earthquake emergency response.

## 5. Population Dynamics and Perceived Impact Area

### 5.1. Population Dynamics

**Figure 5:**
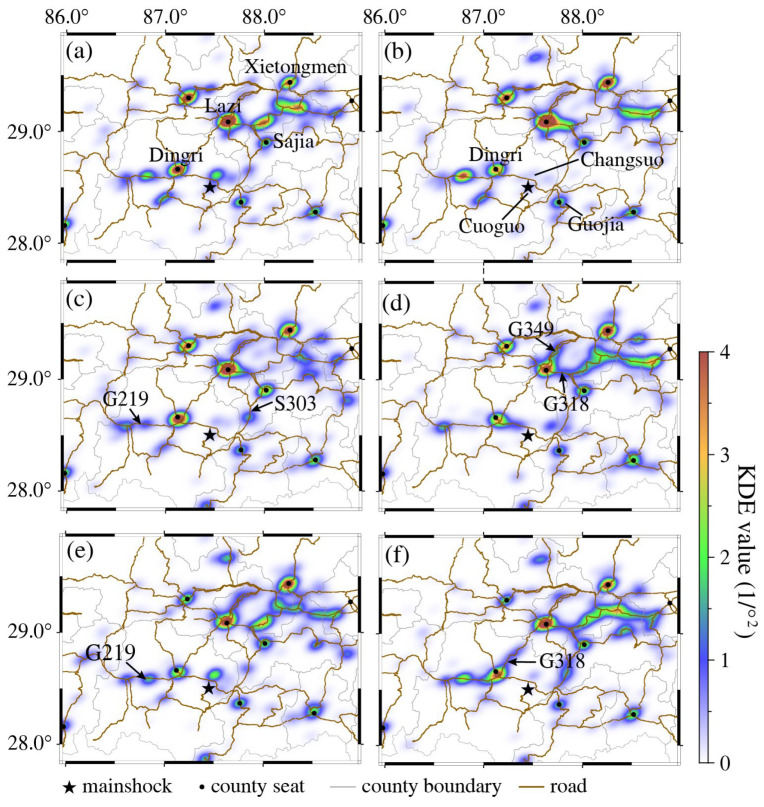
Population dynamics at 30 min intervals. Dynamic population distribution maps were generated from 30 min pre-event to 5.5 h post-event, utilizing active base station data. (**a**) 08:35:16–09:05:16; (**b**) 09:05:16–09:35:16; (**c**) 09:35:16–10:05:16; (**d**) 11:05:16–11:35:16; (**e**) 12:35:16–13:05:16; (**f**) 13:05:16–13:35:16.

Building upon the preceding findings which demonstrate similar spatial distributions across all four indicators (Figure 3), we select active base station data as the representative metric for analyzing post-earthquake population dynamics. We adopt a 14 h observation window (spanning 2 h pre-earthquake to 12 h post-earthquake) and employs KDE to investigate the spatiotemporal evolution of the indicator dataset at minute-level resolutions. To optimize distribution visualization, the bandwidth parameter *h* = 0.08 was selected through iterative testing. Figure 5 illustrates the 30 min-interval population density dynamics from 30 min pre-earthquake to 5.5 h post-earthquake.

The study area demonstrated pronounced population density gradients. High-density clusters dominated northern and eastern regions, particularly at county area (Lazi, Xietongmoin, Sajia, Ngamring county). Low-density areas characterized western and southern zones. Earthquake-adjacent populations primarily aggregated at Changsuo town and Dingri county administrative centers (Figure 5a). Pre-earthquake populations concentrated primarily at county and town administrative centers. Post-earthquake spatial patterns exhibited a “central collapse-peripheral aggregation” configuration. Significant population heat attenuation occurred in earthquake-proximate town (Cuoguo, Changsuo, Quluo town), while distanced Lazi and Xietongmen county (70–130 km), showed sustained heat value increases (Figure 5b).

Significant intensification of population mobility was observed among major residential clusters within 30 min post-earthquake, as evidenced by increased mobile signaling along multiple roadway networks toward the epicentral region. 60 min post-earthquake, characteristic signal migration patterns emerged along highways S303 and G219 (Figure 5c), corresponding to the deployment of first-response units. 120 min post-earthquake, two primary mobilization corridors were identified. G349-oriented movement originated from Xietongmen county and G318-oriented originated from the Kashgar city direction. These trajectories evolved into pronounced linear distributions by 180 min post-earthquake (Figure 5d), with progressive reinforcement of spatial clustering patterns during subsequent phases (Figure 5e). This suggests that emergency resources and teams from various regions converged on the disaster area in a coordinated response effort.

### 5.2. Perceived Impact Area Assessment

**Figure 6:**
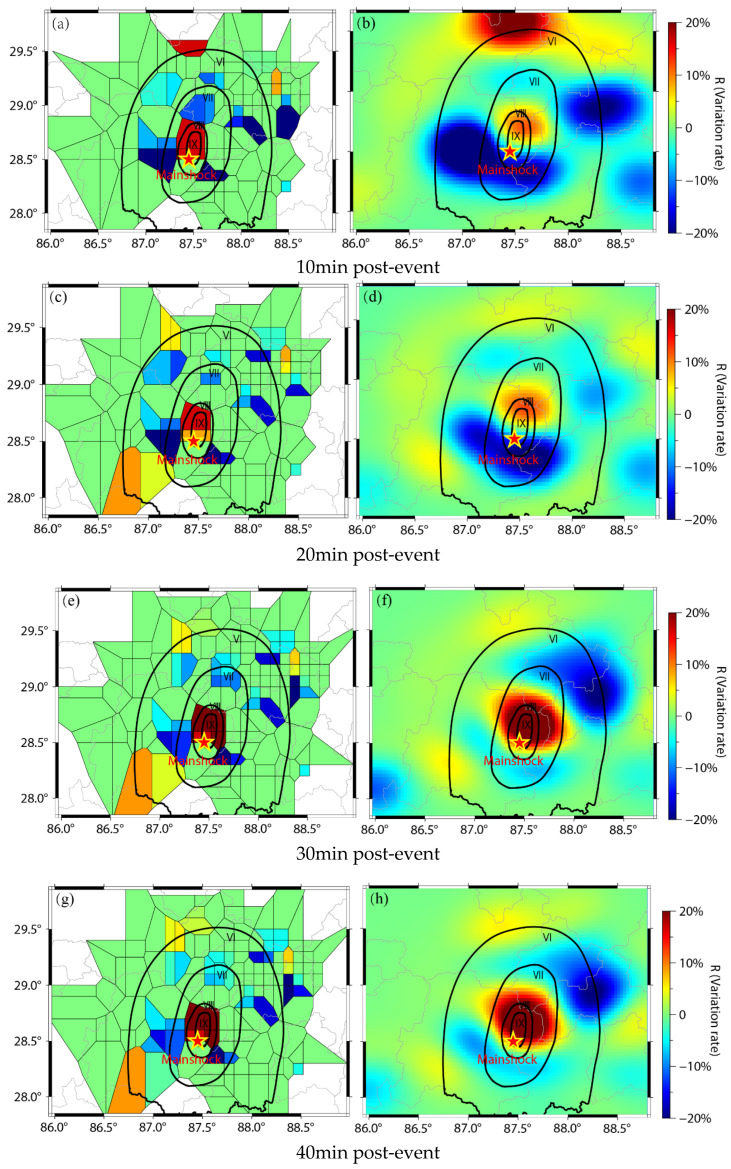
Perceived impact area maps using Thiessen polygon and natural neighbor interpolations. Utilizing active base station data from 10 min to 40 min post-event at 10 min intervals. The black lines represent the isoseismal lines delineating seismic intensity contours. (**a**) Thiessen polygon interpolation 10 min post-event; (**b**) natural neighbor interpolation 10 min post-event; (**c**) Thiessen polygon interpolation 20 min post-event; (**d**) natural neighbor interpolation 20 min post-event; (**e**) Thiessen polygon interpolation 30 min post-event; (**f**) natural neighbor interpolation 30 min post-event; (**g**) Thiessen polygon interpolation 40 min post-event; (**h**) natural neighbor interpolation 40 min post-event. Roman numerals denote macroseismic intensity from degree VI to degree IX.

To achieve rapid post-earthquake impact assessment, we established a pre-event baseline using mobile data from 2 h before the earthquake, then calculated spatiotemporal variation rates (R) at 10-, 20-, 30-, and 40 min intervals post-event using Equation (2). These rates were spatially interpolated using Thiessen Polygon interpolation and Natural Neighbor interpolation (Figure 6). The red areas indicate a downward trend in the rate of change, while the blue corresponds to an upward trend. Color saturation exhibits a positive relationship with the magnitude of variation, where heightened saturation indicates more substantial changes. Specifically, the areas with decreased mobile phone activity (dark red) can intuitively reflect emergency situations such as communication outages or people being trapped. The areas with increased activity (dark blue) reflect the high-frequency mobile phone usage behavior triggered by the earthquake among local residents such as safety confirmation and relatives contact.

High-density urban areas (e.g., county seats) exhibited strong correspondence between interpolated results and empirical intensity surveys (≥VIII area), validating method reliability in data-rich zones. Low-density peripheral regions showed significant discrepancies due to sparse data coverage, highlighting limitations in mountainous terrain. By 40 min post-event, interpolation accuracy stabilized as rescue activities normalized data collection latency. Therefore, within 30 min post-earthquake, the data spanning from 2 h pre-earthquake to 30 min post-earthquake can be utilized to assess the perceived impact area. This approach leverages rapid changes in population mobility, communication signals, and infrastructure dynamics observed during the initial post-seismic phase to delineate regions experiencing significant ground motion or emergency response activation.

The Thiessen Polygon interpolation results demonstrate strong spatial alignment between areas of maximum population activity decline (represented by deep red zones) and the VIII-degree seismic intensity region (Figure 6). According to the China Seismic Intensity Scale, the VIII-degree zone exhibits severe structural damage, including partial collapse of older buildings, indicating high casualty risks and defining it as a strongly perceived impact area. This validates that abrupt post-seismic activity anomalies can serve as critical indicators for identifying high-risk zones requiring prioritized emergency response. For instance, in the case of moderately strong or more destructive earthquakes, base stations and power facilities in the epicentral area are prone to damage, leading to a significant decline in mobile hotspot data—such as the number of Active base stations and Mobile devices—in the affected region. This method enables the direct and rapid identification of such “signal attenuation zones,” thereby providing clear spatial guidance for rescue operations. Areas with a marked drop in post-earthquake hotspot data should be prioritized for emergency response, and it is advisable to dispatch rescue teams or drones promptly for reconnaissance and verification.

In the analysis results, in addition to areas with declining data, there also exist regions showing increased data activity (dark blue). This may indicate a trend of people gathering in relatively safer areas. Such information can be used to monitor post-disaster population movements and identify potential shelter locations. By further integrating data on emergency shelters released by civil affairs and urban planning departments, the reliability of assessments can be enhanced. This, in turn, can assist command centers in more effectively coordinating evacuations, allocating supplies, and deploying rescue resources.

The earthquake’s high-altitude epicenter, situated at an average elevation of 4923 m, presented several distinctive challenges for data collection and analysis. First, the extremely low density of permanent residents and infrastructure in the region resulted in severely limited mobile data availability, hindering real-time monitoring and crowd-sourced data gathering. Second, the use of Geohash grids for spatial indexing led to numerous cells with null values, particularly across remote and inaccessible mountain areas where observations are sparse or absent. And when applied in such a data-scarce context, natural neighbor interpolation exhibited noticeable overfitting. Artifacts emerged especially within zones requiring extrapolation, undermining the reliability of the output surfaces. In comparison, Thiessen polygon methods, though it is simpler, provided more robust and interpretable results under these constrained conditions

## 6. Conclusions and Discussion

This study conducts a spatiotemporal analysis of four mobile location data indicators—active Wi-Fi hotspots, active base stations, mobile devices, and Wi-Fi-connected devices—and preliminarily validates that mobile positioning data can serve as a reference for post-earthquake disaster sensing. By analyzing changes in population distribution before and after the Dingri earthquake, and combining Thiessen polygons and natural neighbor interpolation, the earthquake-affected areas are assessed. Traditional seismic intensity estimation primarily relies on empirical attenuation relationships and instrumental observations, which, while reflecting the spatial distribution of ground shaking intensity, cannot directly capture the actual population response or disaster impact. This paper attempts to detect the real human reactions to an earthquake through real-time changes in pre- and post-earthquake population distribution, thereby providing a “social sensing” dimension to disaster assessment.

1. Mobile location data enables minute-level temporal resolution and kilometer-level spatial resolution for dynamic population distribution visualization. Compared to traditional census data, it offers significantly improved timeliness and spatial resolution over traditional census data and allows for dynamic analysis of population movements. The results presented in this study can be applied during the rapid disaster assessment phase. Within a short period after the earthquake (e.g., from 2 h before to 10 min after the event), areas with abnormal population aggregation, evacuation, or disappearance can be identified to preliminarily infer zones that may have suffered stronger impacts. This approach provides auxiliary reference during the “black-box” period of disaster information, helping emergency managers quickly identify areas with abnormal population changes, initially locate potential hardest-hit zones or evacuation gathering points, and notably assist in detecting “silent disaster areas” that have not yet reported damage. As more mobile data become available over time, during the resource deployment and rescue phase, the data can further be used to optimize the prioritization and routing of rescue forces and material distribution. In the recovery and reconstruction stage, analysis of population return trends can support the assessment of regional recovery progress and long-term resource allocation.
2. All four mobile indicators near the epicenter showed varying degrees of decline post-earthquake, confirming a strong correlation between mobile data and seismic events for accurate perception zone delineation. Comparative interpolation results reveal methodological trade-offs: natural neighbor interpolation produces smoother curves but suffers from overfitting in data-sparse areas, while Thiessen polygons exhibit jagged boundaries. Although both methods lack precision in identifying directional seismic impact fields, analyzing the spatial distribution of mobile phone location data change rates across different regions allows for the generation of a population-impact distribution map, similar in form to a seismic intensity map. While this result differs from traditional intensity maps based on ground-motion parameters, its advantage lies in more directly reflecting the actual effects of an earthquake on human activity and social functioning, rather than merely indicating physical shaking intensity. In rescue decision-making, such a “population impact distribution” can provide valuable reference for resource allocation, offering practical significance particularly in assessing the urgency of rescue needs across different areas. Future improvements should integrate seismic intensity attenuation relationships and instrumental intensity data to refine assessment accuracy for emergency response.
3. Following the earthquake, all four mobile indicators exhibited distinct spatial gradients: within 50 km from epicenter, data declined, reflecting prioritized emergency evacuation actions in high-intensity zones (≥VIII). In the 100–150 km range from the epicenter, results demonstrated intensified communication behaviors (including information-seeking and social coordination) inversely correlated with seismic perception intensity. Future research should incorporate demographic characteristics (age, gender, transient populations, etc.) and behavioral pattern analysis into mobile data analytics to improve post-disaster situational awareness and emergency response strategies.

This study has explored a dynamic assessment method for impact area based on mobile location data and intensity maps. Future research will enhance analyses through multi-source data fusion, incorporating instrument-recorded intensity data and population mortality distribution. By integrating spatiotemporal pattern recognition algorithms and risk assessment models, we will refine the methodology for perceived impact area assessment, improving assessment accuracy. Concurrently, we will further develop rapid regional population mortality assessment models and explore applications of artificial intelligence and machine learning algorithms to unlock the potential of massive mobile positioning data.

## Figures and Tables

**Table 1:** Overview of mobile phone location data.

Field	Description
Signaling Indicators	Active base stations, active Wi-Fi, mobile devices, Wi-Fi-connected devices
Collection Time	Timestamp of data upload
Spatial Resolution	1.2 km × 0.6 km (Geohash6)
Temporal Resolution	1 min

**Table 2:** Sample of mobile location data.

Signaling Indicators	Geohash Code	Date	Time/ HH:MM	Count	Longitude/°E	Latitude/°N
Active Wi-Fi Hotspots	tvnmks	7 January 2025	17:33	29	87.59	29.78
Active Base Stations	tvnn8v	7 January 2025	10:24	3	87.58	29.76
Mobile Devices:	tvq8js	7 January 2025	13:19	9	87.63	29.66
Wi-Fi-Connected Devices	tvnz2m	7 January 2025	13:24	6	87.79	29.89

## Data Availability

The raw data supporting the conclusions of this article will be made available by the authors, upon request and in accordance with the consent of the participants as per the data protection agreement.
